# N-Methyl-D-Aspartate(NMDA) Receptor and Voltage-Gated Potassium Channel (VGKC) Antibody-Associated Encephalitides Presenting as First Episode Acute Psychosis

**DOI:** 10.3389/fpsyt.2019.00913

**Published:** 2019-12-13

**Authors:** Charmaine Tang, Kevin Tan, Geraldine Lim, Lynnette Tan, Kay Yaw Tay, Nagaendran Kandiah, Edimansyah Abdin, Swapna K. Verma

**Affiliations:** ^1^Early Psychosis Intervention Program, Institute of Mental Health, Singapore, Singapore; ^2^Department of Neurology, National Neuroscience Institute, Singapore, Singapore; ^3^Department of Psychological Medicine, Tan Tock Seng Hospital, Singapore, Singapore

**Keywords:** autoimmune encephalitis, immunotherapy, N-methyl-D-aspartate (NMDA) receptor, psychosis, voltage-gated potassium channel (VGKC)

## Abstract

**Aim:** Acute psychosis is not an uncommon presenting feature in immune-mediated encephalitides. Most patients improve if properly diagnosed and treated expediently with immunotherapy. Our study aimed to describe the frequency, clinical spectrum, and long-term outcomes in patients presenting with acute psychosis who have anti-N-methyl-D-aspartate (NMDA) receptor or anti-voltage-gated potassium channel (VGKC) encephalitis.

**Method:** We recruited patients aged 16–50 years presenting with less than 1 month of psychotic and neurological symptoms including cognitive dysfunction, seizures, abnormal movements, and/or autonomic dysfunction.

**Results:** Between September 2011 and October 2013, 60 patients with first episode acute psychosis were screened; 15 were recruited and included for analyses. Four (26.7%) patients were diagnosed with anti-NMDA receptor encephalitis and 1 (6.7%) with anti-VGKC encephalitis. We found that the mean serum white blood cell (WBC) count (12.8 × 10^9^/L ± 4.8 vs. 7.9 × 10^9^/L ± 2.6; p = 0.05) and cerebrospinal fluid WBC count (106 cells/µl ± 101 vs. 8.5 cells/µl ± 18.9; p = 0.05) were higher in positive cases. Certain prodromal features such as fever, headache, confusion, facial dyskinesia, and hypersalivation were also more likely to be present in positive cases. Patients with autoimmune encephalitis also tended to be more unwell, with the majority requiring intensive care, had lower global assessment of functioning scores (30 ± 10 vs. 53.7 ± 21.2, p = 0.09), and were not well enough to complete standard psychiatric and cognitive assessments at presentation.

**Conclusion:** Autoimmune encephalitis is not uncommon in patients with acute psychosis. Elevated WBC counts, certain prodromal features, and a more severe illness at presentation should prompt appropriate evaluation.

## Introduction

Immune-mediated encephalitis typically presents with rapidly progressive short-term memory deficits, seizures, and neuropsychiatric symptoms. A substantial number of patients improve if properly diagnosed and treated with immunotherapy (1). Considerations for an autoimmune basis for schizophrenia and other psychiatric disorders date back to the 1960s and continue to be debated (2–4).

Patients presenting with neuropsychiatric symptoms and have antibodies that bind to cell surface neuronal, glial, or synaptic targets, collectively known as neural surface antibodies (NSAbs), have attracted significant attention among neurologists and psychiatrists (5). These patients have antibodies directed against N-methyl-D-aspartate (NMDA) receptor, voltage-gated potassium channel (VGKC) complex, and its subunits, leucine-rich glioma inactivated 1 (LGI1) and contactin-associated protein like 2 (CASPR2), alpha-amino-3-hydroxy-5-methyl-4-isoxazole-propionic acid (AMPA) receptor, and gamma-aminobutyric acid (GABA) receptor. Their presence raises the possibility both of a causal or disease-modifying role and of clinical improvement with immunotherapy (6).

NMDA receptor encephalitis is an autoimmune disorder in which auto-antibodies target NMDA receptors in the brain, leading to their removal from the synapse. Patients manifest with prominent psychiatric symptoms, in particular psychosis, early in the disease course (7). NMDA receptors are ligand-gated cation channels with crucial roles in synaptic transmission and plasticity. Over-activity of NMDA receptors causing excitotoxicity is a proposed underlying mechanism for epilepsy, dementia, and stroke, whereas low activity produces schizophrenia-like symptoms (8). The illness typically starts off as prodromal symptoms consisting of headache, fever, nausea, vomiting, diarrhea, or upper respiratory tract symptoms, and within a few days to weeks develops into psychiatric symptoms including anxiety, insomnia, fear, grandiosity, hyper-religiosity, mania, and paranoia. The initial phase is usually followed by decreased responsiveness, orofacial dyskinesia, seizure, and autonomic instability (9).

Antibodies against VGKC were first recognized as having a potential pathogenic role in disorders of the central nervous system in 2001 (10). Our understanding has further advanced with the discovery that VGKC antibodies consists of antibodies against the proteins that are complexed with the potassium channel, in particular LGI1 and CASPR2. Antibodies against LGI1 and CASPR2 have been associated with neuropsychiatric features (11). Patients with LGI1-antibodies have a limbic encephalitis, often with hyponatremia, and about half of the patients have typical faciobrachial dystonic seizures. CASPR2-antibodies cause a more variable syndrome of peripheral or central nervous system symptoms, almost exclusively affecting older males (12).

Patients with NMDA receptor and VGKC encephalitis not infrequently present with acute psychosis to psychiatrists first (13–16). Most cases go on to develop neurological symptoms which would then trigger an evaluation for non-psychiatric illness, including infectious, autoimmune, neurodegenerative, and metabolic disorders which may cause psychotic symptoms. An important clinical challenge is identifying these patients in the course of their illness early, so that the selection of those requiring comprehensive evaluation can be rationalized (6, 17). There have been studies examining the prevalence and incidence of autoimmune encephalitis in Western populations. Dubey et al. found that the prevalence and incidence of autoimmune encephalitis was 13.7/100,000 and 0.8/100,000 person-years respectively; the prevalence of NMDA receptor was 0.6/100,000; and the prevalence of LGI1 was 0.7/100,000 (18). Findings from previous studies suggest that there may be a higher prevalence of autoimmune encephalitides among Asians. A cohort of UK patients with NMDA receptor encephalitis described a relatively high proportion (29%) of non-Caucasians (two Chinese and one each from Pakistan, Malaysia, Nigeria, India, Iraq, and Singapore) (19).

In the last few years, there have been a number of studies examining NMDA receptor, VGKC, and other NSAbs autoimmunity in prospectively recruited patients with psychiatric illness, with conflicting results. Some have detected low to moderate proportions of patients with autoantibodies (20–24), while others have none detected even with serum and cerebrospinal fluid (CSF) from banked samples during the acute presentation (25–28). Our study aimed to describe the frequency of NMDA receptor or VGKC encephalitis, the clinical and paraclinical features, and long-term psychiatric, neurological, and cognitive outcomes among patients presenting with the first episode of acute psychosis in Singapore.

## Methods

### Study Design and Participants

Eligible patients presenting with a first episode of psychosis were recruited from the Institute of Mental Health (IMH), a tertiary psychiatric hospital; National Neuroscience Institute (NNI), a neurological center; and Tan Tock Seng Hospital (TTSH), a general hospital, in Singapore, between September 2011 and October 2013. Inclusion criteria were age 16–50 years, the presence of less than 1 month of psychotic symptoms, and any of the following neurological symptoms: cognitive dysfunction, seizures, abnormal movements, or autonomic dysfunction. Patients were excluded if there was alcohol or drug-induced psychosis as determined by clinical history and/or a positive drug screen. Written informed consent was obtained. For cognitively impaired participants or those under 21 years of age, consent was obtained from their legally acceptable representative. The study was approved by the National Healthcare Group Domain Specific Review Board (Ref 2011/00069) and Singhealth Centralized Institutional Review Board (Ref 2011/409/A).

### Procedures

All participants underwent a detailed neurological evaluation and work-up to rule out medical causes of psychosis as part of the routine clinical evaluation. The work-up included blood and CSF laboratory investigations, as well as brain imaging studies. Serum and CSF were tested for NMDA receptor antibodies, and serum was tested for VGKC complex antibodies. NMDA receptor and VGKC complex antibody testing were done at the Weatherall Institute of Molecular Medicine, University of Oxford, United Kingdom. A live cell-based assay for the detection of IgG antibodies binding to the NMDA receptor NR1/NR2b subunits was used. VGKC-complex antibodies were measured using a radioimmunoprecipitation assay. For the study, additional psychiatric, neurological, and cognitive assessments were conducted at the time of presentation (baseline), and at 3-monthly intervals up to 1 year. Psychiatric assessments included the Structured Clinical Interview for DSM-IV-TR (SCID) for diagnoses, Brief Psychiatric Rating Scale (BPRS) for severity of psychopathology, and Global Assessment of Functioning (GAF) scale for assessment of socio-occupational and psychological functioning. The intraclass correlation coefficient for BPRS was 0.96. Neurological assessments included a full neurological examination, Unified Dystonia Rating Scale (UDRS), and Movement Disorder Society—Unified Parkinson’s Disease Rating Scale (MDS-UPDRS) subscale 3 to evaluate abnormal movements. Cognitive assessments included the Mini Mental State Examination (MMSE) and Montreal Cognitive Assessment (MOCA) for global cognition, Frontal Assessment Battery (FAB) and Color Trails Test for executive function, Alzheimer’s Disease Assessment Scale (ADAS-Cog) for episodic memory, and Digit Span for visuomotor speed. The ratings were performed by experienced psychiatrists (LT, SV) and neurologists (KT, KYT, NK) trained in the use of these instruments.

### Statistical Analysis

Data was analyzed using SAS version 9.4 (SAS Institute, Cary, NC). Descriptive analyses were performed to produce mean and standard deviation for continuous variables, and frequency and percentages for categorical variables. Significant mean differences between two groups were tested using the Mann Whitney U test. Statistical significance was set at p < 0.05.

## Results

**Figure 1:**
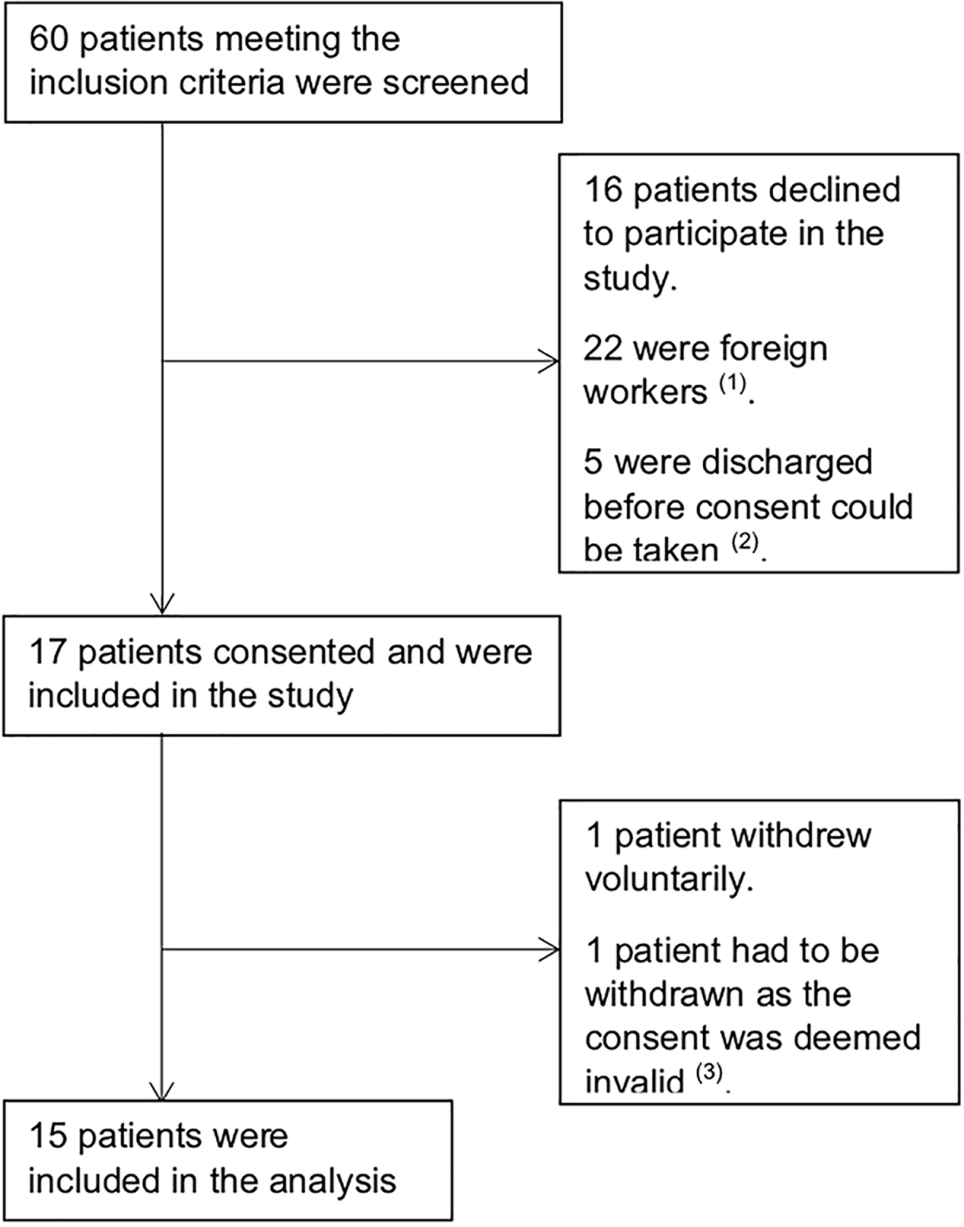
Trial Profile. (1) Foreign workers, typically domestic helpers and laborers, were excluded as they were repatriated back to their home country as soon as clinically stable and long-term follow up would not have been possible. (2) These patients were not able to provide consent as they were unwell and no legally acceptable representative was available. (3) Consent for this patient was obtained after baseline assessments were completed. As such, the authors decided to withdraw the patient’s data from the study.

Between September 2011 and October 2013, 60 patients with first episode acute psychosis meeting the inclusion criteria were screened and 17 consented to be included in the study. One patient later withdrew voluntarily, and another was withdrawn as the consent was deemed invalid; 15 patients were included for analysis (**Figure 1**).

On antibody testing, 4 (26.7%) patients were positive for NMDA receptor antibody and 1 (6.7%) was positive for VGKC complex antibody with a serum titer of 373 pmol/L. All were Chinese females with a mean age of 26.4 (SD = 10.6) years. Of the 10 patients who were negative on antibody testing, 8 (80%) were females, 6 (60%) were Chinese, and 4 (40%) were Malay; the mean age was 29.4 (SD = 5.7) years (**Table 1**).

**Table 1:** Participant characteristics.

	Positive for anti-NMDA receptor or anti-VGKC encephalitis		Total (n = 15)	p value*
	Yes (n = 5)	No (n = 10)		
**Characteristics**				
Age—years, mean (SD)	26.4 (10.6)	29.4 (5.7)	28.4 (7.4)	0.297
Gender—no. (%)				
Female	5 (100)	8 (80.0)	13 (86.7)	.^
Male	0 (0)	2 (20.0)	2 (13.3)	
Ethnicity—no. (%)				
Chinese	5 (100)	6 (60.0)	11 (73.3)	.^
Malay	0 (0)	4 (40.0)	4 (26.7)	
Antibody status—no. (%)				
Anti-NMDA receptor	4 (80.0)	0	4 (26.7)	.^
Anti-VGKC	1 (20.0)	0	1 (6.7)	

*Significance was set at p value < 0.05. ^ Not estimated due to zero cells. NMDA, N-methyl-D-aspartate; VGKC, Voltage gated potassium channels; SD, standard deviation.

### Prodromal Symptoms

Patients were assessed for the presence of the following prodromal symptoms in the preceding 14 days leading up to their first presentation: fatigue, fever, headache, sore throat, nasal discharge, cough, anorexia, nausea, vomiting, diarrhea, confusion, impaired memory, abnormal speech, seizures, facial dyskinesia, arm dyskinesia, leg dyskinesia, rigidity, myoclonus, oculogyric crisis, opisthotonic posture, startle response, tachy- or brady-cardia, labile or elevated blood pressure, hyperhidrosis, hypersalivation, delusion, hallucination, unusual behavior, and personality change. All who were antibody positive had fever; 3 out of 5 (60%) had headache, confusion, facial dyskinesia, and hypersalivation. Of the 10 patients who were antibody negative, 4 (40%) had fever, 1 (10%) headache, 9 (90%) confusion, 1 (10%) facial dyskinesia, and none hypersalivation (**Table 2**).

**Table 2:** Prodromal symptoms.

	Positive for anti-NMDA receptor or anti-VGKC encephalitis	
	Yes (n = 5)	No (n = 10)
**General symptoms**		
Fatigue—no. (%)		
- Yes	0 (0)	2 (20)
- No	5 (100)	6 (60)
- Unknown	0 (0)	2 (20)
Fever—no. (%)		
- Yes	5 (100)	4 (40)
- No	0 (0)	6 (60)
Headache—no. (%)		
- Yes	3 (60)	1 (10)
- No	2 (40)	7 (70)
- Unknown	0 (0)	2 (20)
Sore throat—no. (%)		
- Yes	1 (20)	0 (0)
- No	4 (80)	7 (70)
- Unknown	0 (0)	3 (30)
Nasal discharge—no. (%)		
- Yes	1 (20)	0 (0)
- No	4 (80)	7 (70)
- Unknown	0 (0)	3 (30)
Cough—no. (%)		
- Yes	0 (0)	1 (10)
- No	5 (100)	6 (60)
- Unknown	0 (0)	3 (30)
Anorexia—no. (%)		
- Yes	0 (0)	0 (0)
- No	5 (100)	8 (80)
- Unknown	0 (0)	2 (20)
Nausea—no. (%)		
- Yes	1 (20)	0 (0)
- No	4 (80)	8 (80)
- Unknown	0 (0)	2 (20)
Vomiting—no. (%) - Yes - No - Unknown	1 (20) 4 (80) 0 (0)	0 (0) 8 (80) 2 (20)
Diarrhea—no. (%)		
- Yes	0 (0)	0 (0)
- No	5 (100)	8 (80)
- Unknown	0 (0)	2 (20)
**Cognitive symptoms**		
Confusion—no. (%)		
- Yes	3 (60)	9 (90)
- No	2 (40)	1 (10)
Impaired memory—no. (%)		
- Yes	1 (20)	3 (30)
- No	3 (60)	5 (50)
- Unknown	1 (20)	2 (20)
Abnormal speech—no. (%)		
- Yes	2 (40)	5 (50)
- No	3 (60)	5 (50)
**Movement disorders**		
Facial dyskinesia—no. (%)		
- Yes	3 (60)	1 (10)
- No	2 (40)	9 (90)
Arm dyskinesia—no. (%)		
- Yes	1 (20)	1 (10)
- No	4 (80)	9 (90)
Leg dyskinesia—no. (%)		
- Yes	1 (20)	0 (0)
- No	4 (80)	10 (100)
Rigidity—no. (%)		
- Yes	2 (40)	0 (0)
- No	3 (60)	10 (100)
Myoclonus—no. (%)		
- Yes	0 (0)	0 (0)
- No	5 (100)	10 (100)
Oculogyric crisis—no. (%)		
- Yes	0 (0)	0 (0)
- No	5 (100)	10 (100)
Opisthotonic posture—no. (%)		
- Yes	0 (0)	0 (0)
- No	5 (100)	9 (90)
- Unknown	0 (0)	1 (10)
**Seizures and Neurological symptoms**		
Seizures—no. (%)		
- Yes	0 (0)	0 (0)
- No	5 (100)	10 (100)
Startle response—no. (%)		
- Yes	0 (0)	0 (0)
- No	5 (100)	10 (100)
**Autonomic dysfunction**		
Tachy- or brady-cardia—no. (%)		
- Yes	2 (40)	1 (10)
- No	3 (60)	9 (90)
Labile or elevated blood pressure—no. (%)		
- Yes	1 (20)	1 (10)
- No	4 (80)	9 (90)
Hyperhydrosis—no. (%)		
- Yes	1 (20)	0 (0)
- No	4 (80)	10 (100)
Hypersalivation—no. (%)		
- Yes	3 (60)	0 (0)
- No	2 (40)	10 (100)

NMDA, N-methyl-D-aspartate; VGKC, Voltage gated potassium channels.

### Baseline Psychiatric and Cognitive Assessments

At baseline, only 2 (40%) patients who were antibody positive were clinically well enough for the BPRS assessment to be completed, whereas all patients who were antibody negative completed BPRS assessment. The mean BPRS score was higher for antibody positive than antibody negative patients, but not statistically significant (59.5 ± 9.2 vs. 41.9 ± 18.5; p = 0.16). Baseline GAF was lower for antibody positive than antibody negative cases, but not statistically significant (30 ± 10 vs. 53.7 ± 21.2; p = 0.09). Only 1 (20%) antibody positive patient was able to complete MMSE and MOCA; none were able to complete FAB, Color Trails Test, ADAS-Cog, and Digit Span.

### Baseline Laboratory Investigations

The mean serum white blood cell (WBC) count was higher for antibody positive than antibody negative cases (12.8 × 10^9^/L ± 4.8 vs. 7.9 × 10^9^/L ± 2.6; p = 0.05). The mean CSF WBC count was higher for antibody positive than antibody negative cases (106 cells/µl ± 101 vs. 8.5 cells/µl ± 18.9; p = 0.05) (**Table 3**).

**Table 3:** Baseline laboratory and radiological investigations.

	Positive for anti-NMDA receptor or anti-VGKC encephalitis		p value*
	Yes (n = 5)	No (n = 10)	
Serum WBC count—×10^9^, mean (SD)	12.8 (4.8)	7.9 (2.6)	0.05
CSF WBC count—cells/µl, mean (SD)	106 (101)	8.5 (18.9)	0.05
***Imaging findings for positive cases***			
Patient 1: (NMDA +) Non-contrast CT Brain and MRI Brain did not reveal any significant abnormalities; Pelvic imaging (Ultrasound and MRI) suggested a right ovarian teratoma. Patient 2 (NMDA +): Non-contrast CT Brain revealed diffuse cerebral edema; Pelvic imaging (Ultrasound and MRI) suggested a right ovarian teratoma. Patient 3 (NMDA +): Non-contrast CT Brain and MRI Brain with contrast did not reveal any significant abnormalities; Pelvic imaging was not done. Patient 4 (NMDA +): MRI Brain with contrast revealed mild leptomeningeal enhancement; Electroencephalography showed severe diffuse encephalopathy; Abdomino-pelvic CT did not reveal any significant abnormalities. Patient 5 (VGKC +): CT and MRI Brain with contrast did not reveal any significant abnormalities. Electroencephalography did not reveal any significant abnormalities.			

*Significance was set at p value < 0.05. ^ Not estimated due to zero cells. CSF, Cerebrospinal fluid; CT, Computed tomography; MRI, Magnetic resonance imaging; NMDA, N-methyl-D-aspartate; VGKC, Voltage gated potassium channels; WBC, White blood cell; SD, standard deviation.

### Baseline Radiological Investigations

All antibody positive cases had a baseline brain imaging study done as part of their clinical evaluation. One patient had only computed tomography (CT) scan; one patient had only magnetic resonance imaging (MRI) scan; three patients had both CT and MRI scan. One patient had diffuse cerebral edema (CT scan); another’s scan revealed mild leptomeningeal enhancement (MRI scan). None of the brain imaging scans performed on antibody negative cases revealed any abnormalities. Three of four patients with NMDA receptor encephalitis had abdominal and pelvic imaging (CT, MRI or ultrasound)—two revealed ovarian pathology (**Table 3**).

### Case Vignettes With Clinical Course, Treatment, and Outcomes

All patients who were antibody positive presented with non-specific viral-like symptoms and later developed psycho-behavioral symptoms. Three (60%) antibody positive cases required intubation and management in the intensive care unit (ICU), whereas none of the negative cases required intubation or ICU care.

Patient 1 (P1) presented with disorganized speech, disorientation, and severe agitation which required management with rapid tranquilizers and restraints. P1’s Glasgow Coma Scale (GCS) score deteriorated to 3 and developed orofacial and limb dyskinesia, and autonomic instability within a few days of the initial presentation. P1 was intubated and managed in ICU where she was initially treated with intravenous ceftriaxone and acyclovir. Brain imaging did not reveal any abnormalities. Pelvic imaging revealed right ovarian teratoma. Both serum and CSF were positive for NMDA receptor antibodies. P1 was treated with a course of intravenous methylprednisolone, intravenous immunoglobulins, and underwent an oophorectomy. P1 continued to deteriorate and further underwent plasma exchange. P1 was also treated with clonazepam, benzhexol, amantadine, tetrabenazine, intravenous midazolam, and propofol for severe dyskinesia. Despite aggressive treatment, P1 eventually required extracorporeal membrane oxygenation (ECMO) for prolonged cardiac and respiratory support. P1 remained ill throughout her ICU stay; a few months after her initial presentation, a joint decision was made by her family and the care team to withdraw life support.

Patient 2 (P2) presented with irrelevant and pressured speech, disorganized behaviors (talking to oneself, dancing inappropriately), auditory hallucinations, and emotional lability. P2 was initially diagnosed with bipolar I disorder with psychotic features and treated with risperidone. P2 developed autonomic instability, swinging pyrexia, and dyskinesia, and the GCS score deteriorated to 6 within days of initial presentation. P2 was intubated and initially treated with intravenous ceftriaxone and acyclovir. CT brain revealed diffuse cerebral edema; P2 was treated with intravenous mannitol and had an external ventricular drain inserted. Pelvic imaging revealed right ovarian teratoma. Both serum and CSF were positive for NMDA receptor antibodies. P2 was treated with a course of intravenous methylprednisolone, intravenous immunoglobulins, and underwent an oophorectomy. P2 gradually showed signs of neurological improvement after a period of ICU stay and rehabilitation. At 1 year follow up, P2 did not have any psychopathology, was back to the premorbid level of functioning, and did not have any neurological or cognitive deficits.

Patient 3 (P3) presented with generalized anxiety, insomnia, micropsia, agitation, oropharyngeal dyskinesia, disorganized speech, and fluctuating levels of consciousness. P3 reported amenorrhea for several months prior to her presentation. At presentation, there was a high index of suspicion for NMDA receptor encephalitis; P3 was treated with a course of intravenous methylprednisolone, as well as intravenous ceftriaxone and acyclovir. Both CT and MRI brain imaging did not reveal any abnormalities. Pelvic imaging was not done. P3 had improved orientation, stable parameters, and resolution of dyskinesia a few days after admission. Both serum and CSF later returned positive for NMDA receptor antibodies. At 3 months follow up, P3 did not have any psychopathology, was back to the premorbid level of functioning, and did not have any neurological or cognitive deficits.

Patient 4 (P4) presented with anxiety, depressed mood, emotional lability, insomnia, visual hallucinations (seeing images of her face everywhere), and disorganized behaviors (talking to herself). P4 developed dystonia, oral and limb dyskinesia, had autonomic instability and a drop in the GCS to 11 (spontaneous eye opening; confused speech; able to localize pain). MRI brain with contrast showed mild leptomeningeal enhancement; electroencephalography (EEG) showed severe diffuse encephalopathy. Abdomino-pelvic CT did not reveal any pathology. P4 was initially treated with intravenous ceftriaxone, acyclovir, and sodium valproate. Serology was positive for NMDA receptor antibodies. P4 was treated with a course of intravenous methylprednisolone and intravenous immunoglobulins. However, P4 deteriorated and had to be intubated, treated with intravenous rituximab, and also given clonazepam, haloperidol, benserazide, and intravenous midazolam for worsening dystonia and dyskinesia. P4 eventually stabilized and was given maintenance immunotherapy with azathioprine and underwent several months of rehabilitation.

Patient 5 had VGKC encephalitis and has been separately described in our previous case report (29).

## Discussion

Our study is the first to examine the frequency and baseline characteristics of patients in an Asian population with NMDA receptor or VGKC encephalitis presenting with acute psychosis. We have described the clinical presentation, course, treatment, and outcomes of these patients which add to the growing body of literature on autoimmune encephalitides. We have also described some of the key features early in the course of the presentation which may alert clinicians to consider immune-mediated limbic encephalitis as a differential diagnosis, so as to rationalize when we should do further evaluations for non-psychiatric causes of acute psychosis.

We found that 4 (26.7%) of 15 participants had anti-NMDA receptor encephalitis and 1 (6.7%) had anti-VGKC encephalitis. Considering that 5 (8.3%) out of the 60 patients who were screened were eventually diagnosed with an autoimmune encephalitis, our study suggests that the condition is much more common than originally thought and clinicians would do well to have a high index of suspicion when assessing patients with first-episode psychosis (FEP), particular if the presentation is acute and neurological symptoms (i.e. cognitive dysfunction, seizures, abnormal movements, or autonomic dysfunction) are present. In a recent study, Lennox and colleagues found that 9% of patients with FEP had serum antibodies against one or more of the neuronal cell surface antigens compared with 4% of controls (13). There may be a role for systematic screening of current identified autoantibodies to neuronal cell surface antigens in patients with psychotic disorders to characterize the prevalence of immune-mediated encephalitis among these patients, with implications for diagnosis, prognosis, and treatment.

Estimates of NSAb prevalence in primary psychotic disorders have varied largely with symptom duration and assay approaches, but generally studies of early disease with live cell-based assays have detected NMDA receptor antibodies at higher rates than controls (25), whereas those of chronic disease using commercial fixed cell-based assays have been negative (30, 31).

Our study had several limitations. The sample size was small and we were not able to obtain informed consent from the patient or their legally acceptable representative in 71.7% of the patients we screened. This was likely because the patients were acutely unwell and hence less inclined to participate in a research study. There was also a significant amount of missing data as the positive cases were too unwell to complete the baseline psychiatric, neurological, and cognitive assessments.

Despite the small sample size, we found that the mean serum and CSF WBC counts were higher in those who were positive on antibody testing. We also found that certain signs and symptoms at presentation, such as fever, headache, confusion, facial dyskinesia, and hypersalivation should raise the suspicion of autoimmune encephalitis. Lastly, patients with autoimmune encephalitis tend to be clinically more unwell at baseline, with the majority requiring intubation, ICU care, had lower GAF scores, and were not able to complete standard psychiatric and cognitive assessments. Notably, the study population is not representative of a typical case load in an early psychosis intervention team. We recruited patients who present with less than a month of psychotic symptoms with at least a neurological feature. The results from our study seem to support focused screening of those with certain clinical features rather than blanket screening of all patients with first episode psychosis.

## Data Availability Statement

The datasets generated for this study are available on request to the corresponding author.

## Ethics Statement

This study was carried out in accordance with the recommendations of the National Healthcare Group Domain Specific Review Board (Ref 2011/00069) and Singhealth Centralized Institutional Review Board (Ref 2011/409/A) with written informed consent from all subjects. All subjects gave written informed consent in accordance with the Declaration of Helsinki. The protocol was approved by the National Healthcare Group Domain Specific Review Board (Ref 2011/00069) and Singhealth Centralized Institutional Review Board (Ref 2011/409/A).

## Author Contributions

All authors were involved in the drafting and review of the manuscript, participated in the discussion on the topic, and had approved the version of submitted manuscript.

## Funding

The study was supported by the Singapore National Healthcare Group Small Innovative Grant (NHG-SIG) which provided funding of SGD 100,000 between April 2011 and October 2013. The funding source was not involved in the design and conduct of the study; analysis and interpretation of the data; and preparation, review, or approval of the manuscript.

## Conflict of Interest

KT has received travel grants and compensation from Novartis, Merck, UCB, Sanofi, and Eisai for consulting services.

The remaining authors declare that the research was conducted in the absence of any commercial or financial relationships that could be construed as a potential conflict of interest.
